# Loss of MYO5B expression deregulates late endosome size which hinders mitotic spindle orientation

**DOI:** 10.1371/journal.pbio.3000531

**Published:** 2019-11-04

**Authors:** Changsen Leng, Arend W. Overeem, Fernando Cartón-Garcia, Qinghong Li, Karin Klappe, Jeroen Kuipers, Yingying Cui, Inge S. Zuhorn, Diego Arango, Sven C. D. van IJzendoorn

**Affiliations:** 1 Department of Biomedical Sciences of Cells and Systems, section Molecular Cell Biology, University of Groningen, University Medical Center Groningen, Groningen, the Netherlands; 2 Group of Biomedical Research in Digestive Tract Tumors, CIBBIM-Nanomedicine, Vall d'Hebron Research Institute (VHIR), Universitat Autònoma de Barcelona (UAB), Barcelona, Spain; 3 Department of Gastroenterology and Hepatology, University of Groningen, University Medical Center Groningen, Groningen, the Netherlands; 4 Department of Biomedical Engineering, University of Groningen, University Medical Center Groningen, Groningen, the Netherlands; Utrecht University, NETHERLANDS

## Abstract

Recycling endosomes regulate plasma membrane recycling. Recently, recycling endosome–associated proteins have been implicated in the positioning and orientation of the mitotic spindle and cytokinesis. Loss of *MYO5B*, encoding the recycling endosome–associated myosin Vb, is associated with tumor development and tissue architecture defects in the gastrointestinal tract. Whether loss of *MYO5B* expression affects mitosis is not known. Here, we demonstrate that loss of *MYO5B* expression delayed cytokinesis, perturbed mitotic spindle orientation, led to the misorientation of the plane of cell division during the course of mitosis, and resulted in the delamination of epithelial cells. Remarkably, the effects on spindle orientation, but not cytokinesis, were a direct consequence of physical hindrance by giant late endosomes, which were formed in a chloride channel–sensitive manner concomitant with a redistribution of chloride channels from the cell periphery to late endosomes upon loss of *MYO5B*. Rab7 availability was identified as a limiting factor for the development of giant late endosomes. In accordance, increasing rab7 availability corrected mitotic spindle misorientation and cell delamination in cells lacking *MYO5B* expression. In conclusion, we identified a novel role for *MYO5B* in the regulation of late endosome size control and identify the inability to control late endosome size as an unexpected novel mechanism underlying defects in cell division orientation and epithelial architecture.

## Abstract 2

Loss of the recycling endosome-associated motor protein myosin Vb causes the formation of giant late endo-lysosomes; these in turn hinder the orientation of the mitotic spindle and chromosome segregation. Deregulated endosome size thus hampers faithful cell division.

## Introduction

Recycling endosomes, strategically positioned at the intersection of endocytic and biosynthetic trafficking pathways, are membrane compartments with versatile functions [1,2]. Recycling endosomes are important for, among others, epithelial cell polarity development, cell-cell adhesion, and cell fate determination [2]. Mutations in recycling endosome–associated and *MYO5B*-encoded myosin Vb protein can cause progressive familial intrahepatic cholestasis [3] and/or microvillus inclusion disease (MVID) [4–6]. MVID phenotypes are the result of defective plasma membrane recycling in intestinal epithelial cells and intestinal tissue architecture defects. Of interest, loss of *MYO5B* expression is also associated with gastrointestinal cancer [7,8] and is a strong prognostic factor for colorectal cancer recurrence [9].

Recycling endosomes and therewith associated proteins, notably the small GTPase rab11a, have also been implicated in mitotic cell division processes including cytokinesis [10,11] and, more recently, the organization and orientation of the mitotic spindle apparatus [12–14]. Mitotic spindle orientation plays a key role in the development and maintenance of epithelial tissue architecture and may function in tumor suppression [15–18]. Defects in mitotic spindle and cell division orientation can cause the mispositioning of cells out of the epithelial layer known as cell delamination [19]. Delamination can cause apoptosis or loss of epithelial cell polarity and has been associated with tumorigenesis [18]. Notably, although myosin Vb is a recycling endosome–associated protein and implicated in cell polarity, cancer, and tissue architecture defects, the role of myosin Vb in mitotic cell division has not been addressed.

In this study, we have investigated mitotic cell division following the loss of *MYO5B* expression in intestinal epithelial cells. It is shown that the loss of *MYO5B* resulted in delayed cytokinesis, perturbed mitotic spindle orientation, and led to the misorientation of the plane of cell division during the course of mitosis and resulted in the delamination of epithelial cells. Mechanistically, we show that loss of *MYO5B* expression, in addition to its effects on plasma membrane homeostasis, also affected late endosome homeostasis and size. Surprisingly, we found that the presence of giant late endosomes that formed upon the loss of *MYO5B* was wholly responsible for mitotic spindle misorientation. These findings add an unexpected new dimension to the role of membrane trafficking proteins in processes that ensure proper mitotic progression and cell division.

## Results

### Loss of *MYO5B* causes mitotic spindle orientation defects and cytokinesis delay

**Fig 1:**
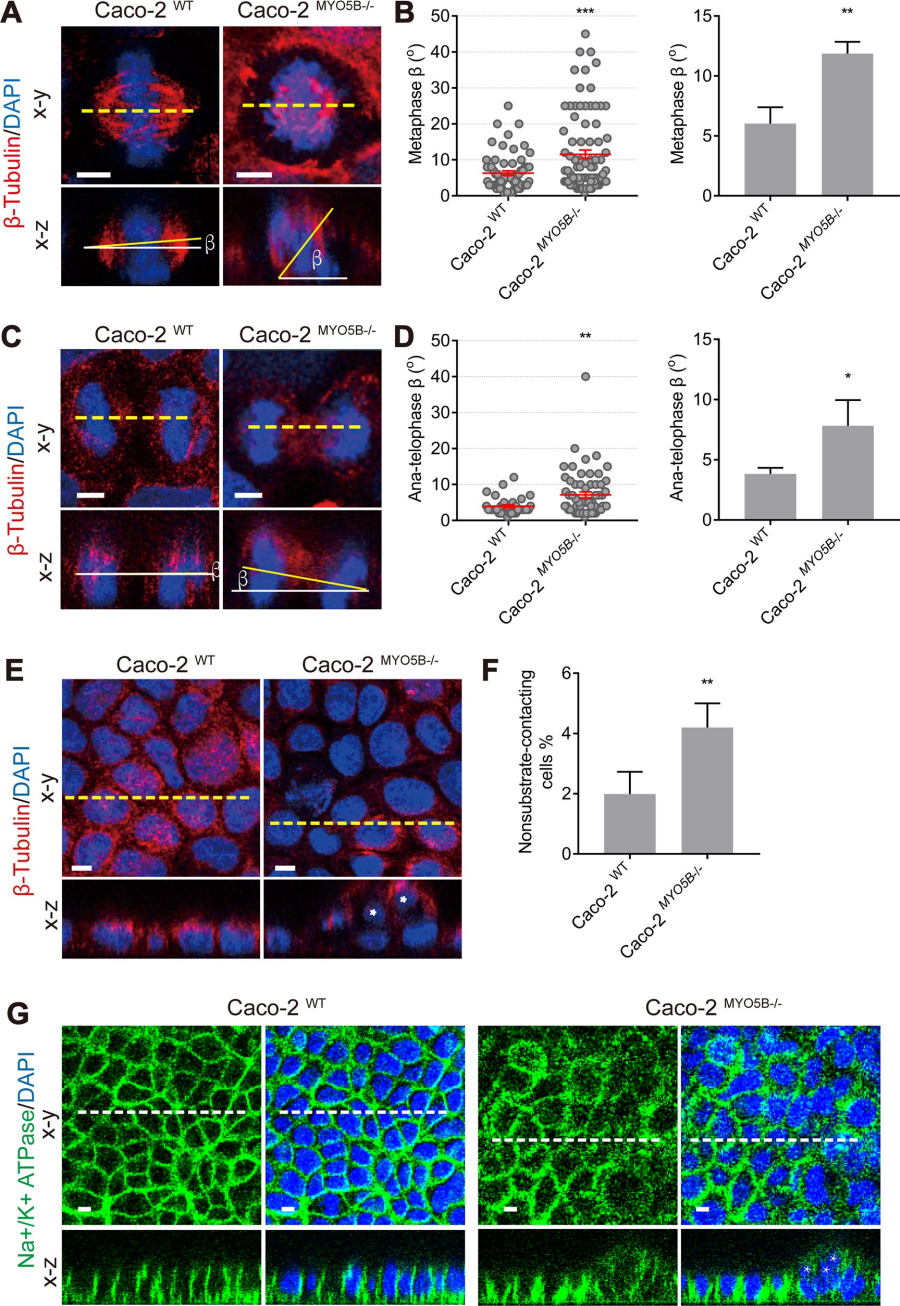
Loss of MYO5B causes mitotic spindle orientation defects. (A) Caco2^WT^ and Caco2^MYO5B−/−^cells in metaphase were fixed and stained as indicated. The β-angle represents the angle between the spindle axis and the substratum in the confocal x-z dimension. (B) The β-angle was quantified in metaphase. (Left side graph) Each dot indicates one cell’s β-angle. (Right side graph) The statistical analysis of the mean for each experiment. *n* ≥ 15 mitotic cells/experiment were analyzed for *N* = 3 independent experiments. Values for each data point can be found in S1 Data. (C) Caco2^WT^ and Caco2^MYO5B−/−^ cells in anatelophase were fixed and stained as indicated. (D) The quantification of β-angle in anatelophase. *n* ≥ 9 mitotic cells/experiment were analyzed for *N* = 3 independent experiments. Values for each data point can be found in S1 Data. (E–F) The presence of cells not contacting the substratum was indicated by asterisks in nuclei (E). The percentage of nonsubstrate-contacting cells was quantified. Values for each data point can be found in S1 Data (F). (G) Na^+^/K^+^ ATPase staining shows basolateral localization in substrate contacting cells but not in multilayered cells. *N* = 3 independent experiments. *t* test,*p < 0.05, **p < 0.01, ***p < 0.001. Error bars indicate ± SEM (dot graph) or + SD (bar graph). Scale bars: 5 μm.

In order to investigate the effects of loss of *MYO5B* on mitotic spindle orientation, we generated *MYO5B* knockout human intestinal epithelial Caco2 cells using CRISPR-Cas9 technology. Knockout of the *MYO5B* gene was verified by the absence of the encoded myosin Vb protein on Western blot (see S1A Fig) and supported by sequencing of the *MYO5B* gene, which revealed the introduction of a premature termination codon in exon 3 (see S1B Fig). *MYO5B* knockout cells (hereafter referred to as Caco2^MYO5B−/−^) and *MYO5B* wild-type (WT) cells (hereafter referred to as Caco2^WT^) were cultured on coverslips and transfected with mCherry-tagged Histone2B to visualize chromosomes and GFP-tagged β-tubulin or, alternatively, labeled with antibodies against these proteins in order to visualize the mitotic spindle apparatus by laser scanning confocal fluorescence microscopy. The orientation of the mitotic spindle relative to the substratum (x-z direction) in cell monolayers was determined in 3D-reconstructed confocal images(x-y direction) of fixed cells by measuring the angle (β) between a line drawn through the spindle poles and a line parallel to the substratum (Fig 1A). The results show that metaphase Caco2^WT^ cells showed an average β-angle of 6° relative to the substratum, and a β-angle beyond 20° was rarely observed (Fig 1B). In contrast, metaphase Caco2^MYO5B−/−^ cells showed an average β-angle of 12° and a significantly higher percentage of cells displayed β-angles beyond 20° and up to 40° (Fig 1A, Fig 1B). This increase in the frequency of tilted mitotic spindles in metaphase Caco2^MYO5B−/−^ cells persisted, albeit to a lesser extent, through subsequent anaphase (Fig 1C, Fig 1D). Aberrant spindle tilting can result in the delamination of epithelial cells from the monolayer and loss of epithelial polarity of delaminated cells [15–17,19,20]. In accordance, in comparison to Caco2^WT^ cells, Caco2^MYO5B−/−^ cell cultures showed an increased percentage of cells that were placed on top of the monolayer (Fig 1E, Fig 1F) and which had lost basolateral surface polarity as evidenced by the nonpolarized distribution of the basolateral resident Na^+^/K^+^-ATPase (Fig 1G). Similar results were obtained when the cells were plated as a monolayer on semipermeable Transwell filter supports (see S2 Fig).

**Fig 2:**
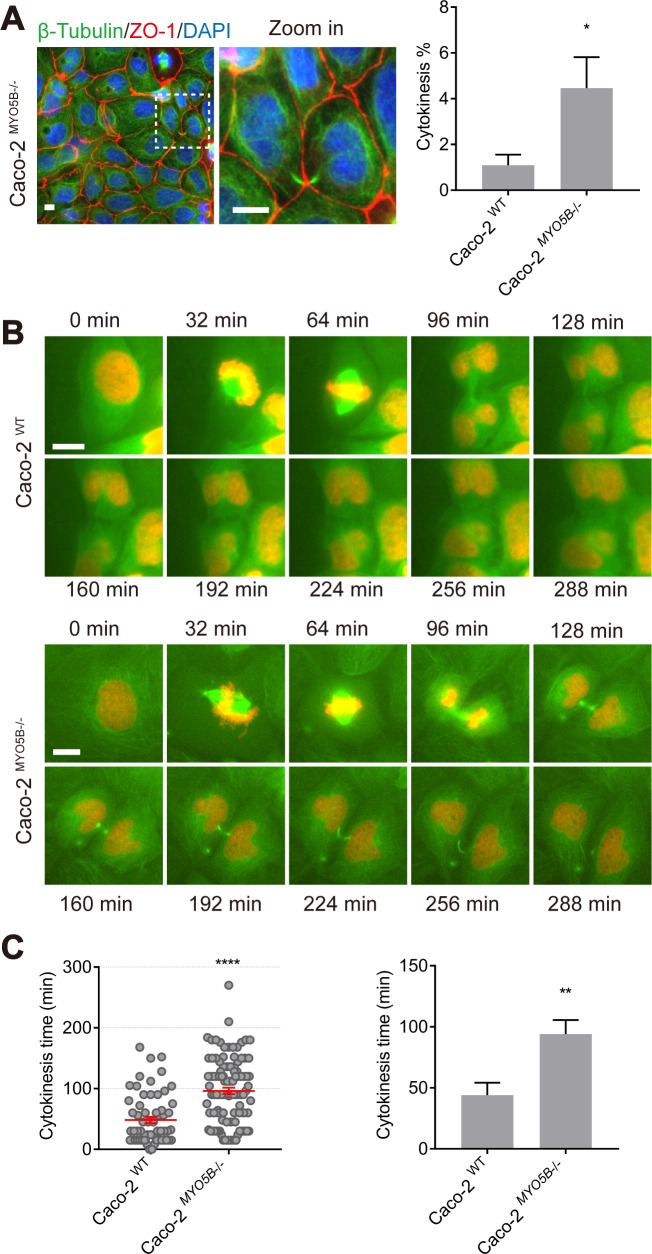
Loss of MYO5B causes cytokinesis delay. (A) Caco2^WT^ and Caco2^MYO5B−/−^cells were fixed and stained as indicated. The percentage of cytokinesis with both daughter cells was quantified. *n* > 1,000 cells/experiment were analyzed for *N* = 3 independent experiments. Values for each data point can be found in S2 Data. (B) Live cell imaging shows x-y-t time-lapse mitosis images on Caco2^WT^ and Caco2^MYO5B−/−^ cells expressing β-tubulin-GFP and histone2B-mCherry. (C) The time of cytokinesis duration was quantified. (Left side graph) Each dot indicates 1 mitotic cell’s cytokinesis time. (Right side graph) The statistical analysis of the mean for each experiment. *n* ≥ 20 cells/experiment were analyzed for *N* = 3 independent experiments. Values for each data point can be found in S2 Data. *t* test, *p < 0.05, **p < 0.01, ****p < 0.0001. Error bars indicate ± SEM (dot graph) or + SD (bar graph). Scale bars: 5 μm.

In addition to the tilted spindle phenotype, Caco2^MYO5B−/−^ cells showed a higher cytokinesis index when compared with Caco2^WT^ cells, as evidenced by the presence of a β-tubulin-positive cytokinesis bridge (Fig 2A). Live cell imaging of mCherry-H2B/GFP-β-tubulin-expressing cells revealed that cytokinesis was delayed in Caco2^MYO5B−/−^ cells (Fig 2B, see S1 Video, S2 Video). Thus, measuring the time that passed from the first appearance until the disappearance of the GFP-β-tubulin-positive cytoplasmic bridge showed an on average 2-fold increase (from 40 to 90 min in Caco2^WT^ and Caco2^MYO5B−/−^ cells, respectively) in cytokinesis duration (Fig 2C).

In conclusion, mitotic spindle orientation defects, and cytokinesis delays were observed in Caco2 cells lacking *MYO5B* expression.

### Concurrence of mitotic spindle orientation defects and the presence of large vacuoles in *MYO5B*-depleted cells

**Fig 3:**
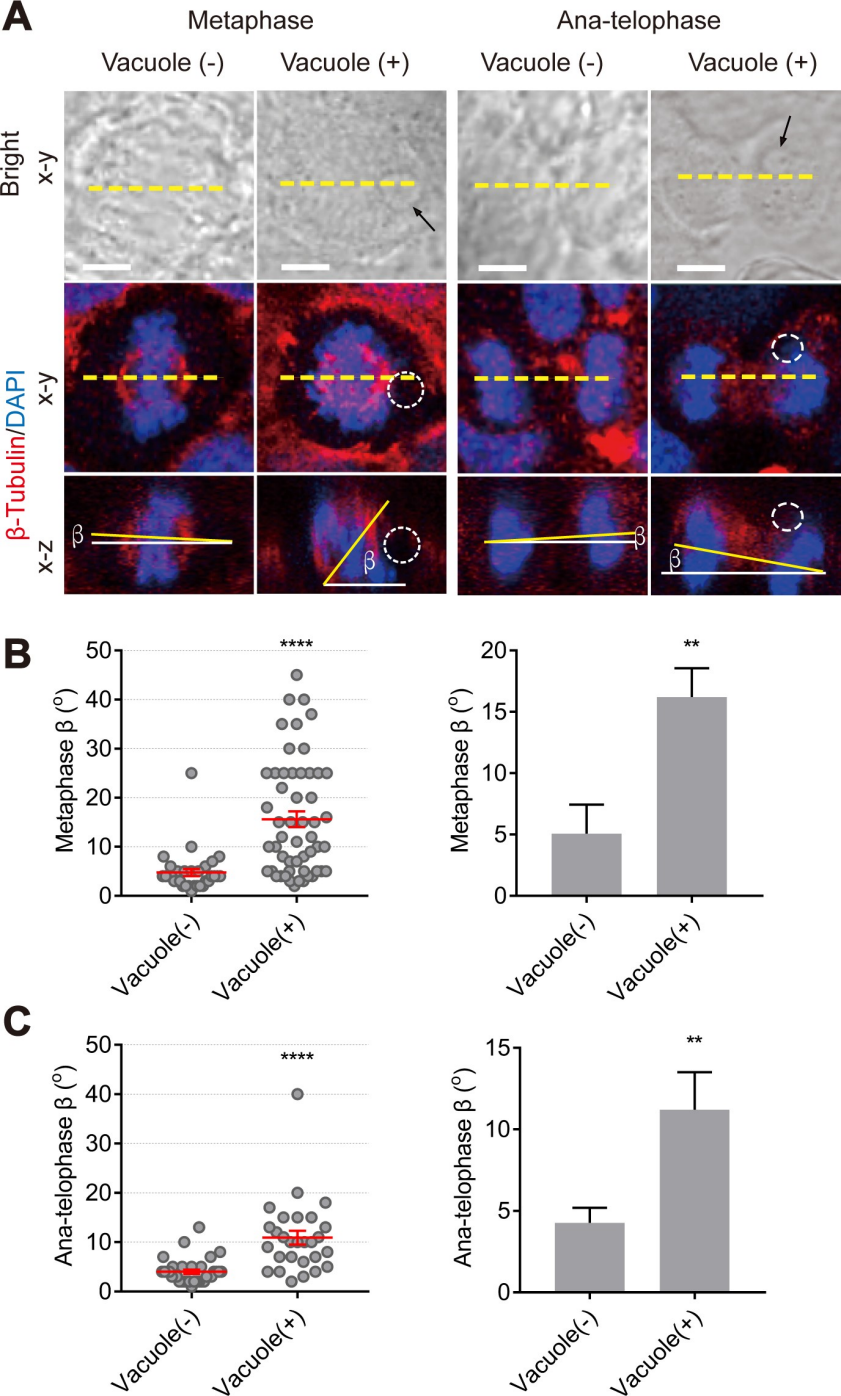
Mitotic spindle orientation defects correlate with the presence of large vacuoles in MYO5B-depleted cells. (A) Caco2^MYO5B−/−^ cells were fixed and stained as indicated. Arrows in bright field and dotted circles in fluorescent field indicate large vacuoles in Caco2^MYO5B−/−^ cells during metaphase and ana-telophase. (B) The β-angle in Caco2^MYO5B−/−^ cells with vacuole and without vacuole during metaphase was quantified. (Left side graph) Each dot indicates one mitotic cell’s metaphase β-angle. (Right side graph) The statistical analysis of the mean for each experiment. *n* ≥ 8 mitotic cells/experiment were analyzed for *N* = 3 independent experiments. Values for each data point can be found in S3 Data. (C) The quantification of β-angle in Caco2^MYO5B−/−^ cells with vacuole and without vacuole during anatelophase. *n* ≥ 6 cells/experiment were analyzed for *N* = 3 independent experiments. Values for each data point can be found in S3 Data. *t* test, **p < 0.01, ****p < 0.0001. Error bars indicate ± SEM (dot graph) or + SD (bar graph). Scale bars: 5 μm.

Upon closer microscopical inspection of cells with tilted mitotic spindles, we noticed that virtually all Caco2^MYO5B−/−^ cells with tilted spindles contained one or more large (i.e., Ø >1 μm) vacuoles (Fig 3A, arrows and dotted white circles [note that this is the same image as in Fig 1A]). Subsequent comparison of the β-angles in Caco2^MYO5B−/−^ cells with a vacuole or without a vacuole revealed that the increase in spindle tilting during metaphase and anaphase was restricted to those Caco2^MYO5B−/−^ cells that displayed a vacuole (Fig 3B, Fig 3C). The absence of a spindle orientation defect in the small intestine of *Myo5b* knockout mice (see S3 Fig), which do form vacuoles but in villus enterocytes and not in the proliferative crypt cells (see below), supports that the mere loss of *Myo5b* expression (i.e., without a vacuole present) did not cause the spindle misorientation phenotype.

**Fig 4:**
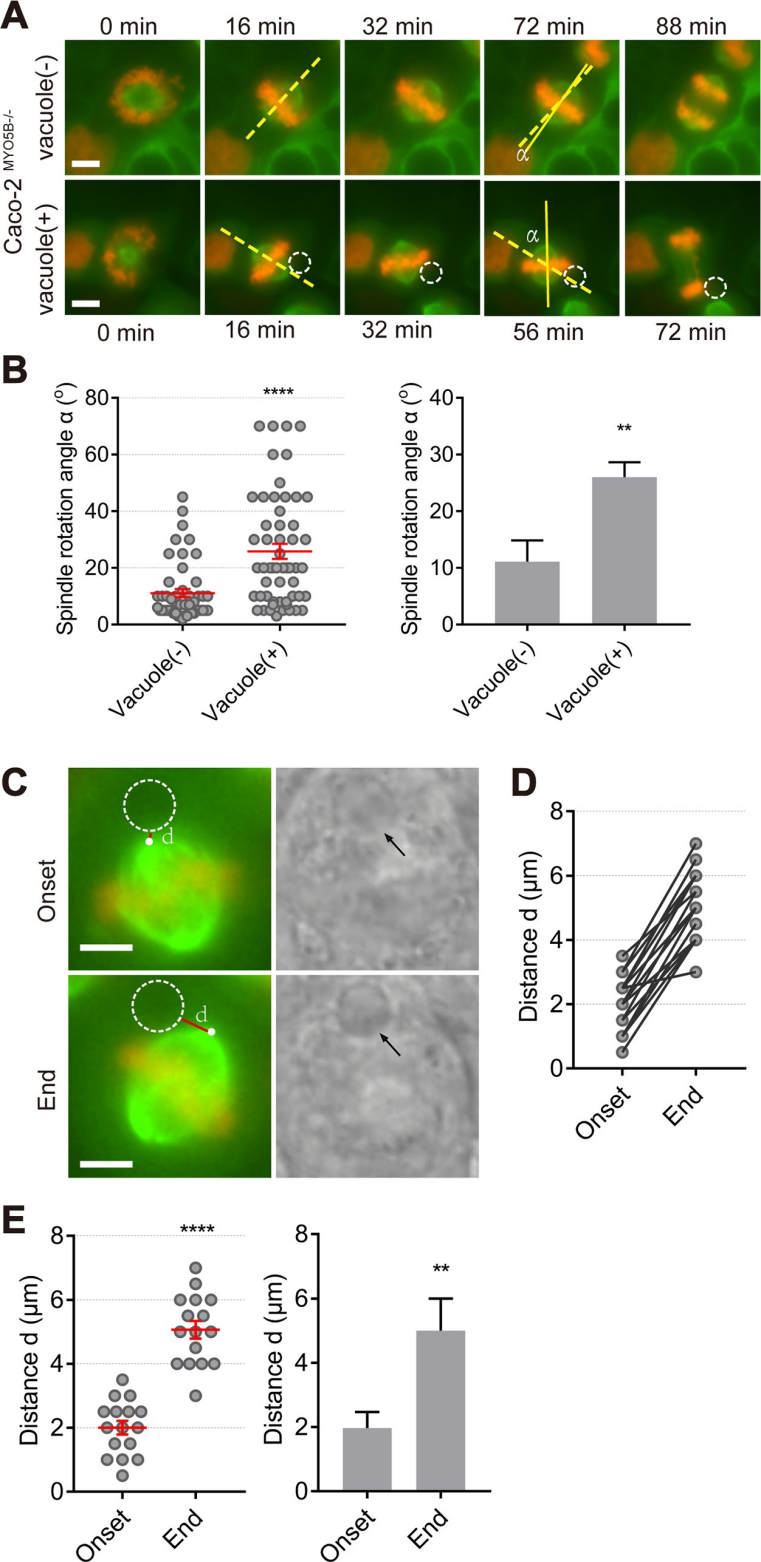
Mitotic spindle orientation defects correlated to the presence of large vacuoles in MYO5B-depleted cells. (A) Live cell imaging shows mitosis images on Caco2^MYO5B−/−^cells expressing β-tubulin-GFP and histone2B-mCherry. The α angle represents the angle between a line drawn through the spindle axis at the onset of metaphase (dotted line) and the end of metaphase (solid line). Dotted circles indicate large vacuoles which were confirmed by bright field. (B) The α angle in Caco2^MYO5B−/−^cells with vacuole and without vacuole during metaphase was quantified. Each dot indicating 1 mitotic cell’s spindle rotation α angle (left). The statistical analysis of the mean for each experiment (right). *n* > 15 cells/experiment were analyzed for *N* = 3 independent experiments. Values for each data point can be found in S4 Data. (C) The position of mitotic spindle relative to vacuole during onset and end of metaphase in Caco2^MYO5B−/−^ cells was quantified by measuring the distance from spindle pole to vacuole (d, red line). Arrows in the bright field and dotted circles in the fluorescent field indicate large vacuoles. (D) The comparison of the distance d in the onset and end of metaphase in Caco2^MYO5B−/−^ cells. Values for each data point can be found in S4 Data. (E) The quantification of the distance d in vacuolated Caco2^MYO5B−/−^ cells. (Left side graph) Each dot indicates one cell’s distance d between the onset and end of metaphase. (Right side graph) The statistical analysis of the mean for each experiment. *n* ≥ 5 cells/experiment were analyzed for *N* = 3 independent experiments. Values for each data point can be found in S4 Data. *t* test, **p < 0.01, ****p < 0.0001. Error bars indicate ± SEM (dot graph) or + SD (bar graph). Scale bars: 5 μm.

We then subjected Caco2^MYO5B−/−^ cells to live cell fluorescence imaging in the x-y plane to investigate the effect of the vacuole on spindle orientation dynamics in real time. In Caco2^MYO5B−/−^ cells without a vacuole, spindle rotation dynamics were minimal during metaphase (Fig 4A, see S3 Video). Thus, the average angle (α) between a line drawn through the spindle axis at the onset of metaphase and the end of metaphase (i.e., the onset of anaphase) was approximately 10°. By contrast, in Caco2^MYO5B−/−^ cells that contained a vacuole, the average α-angle was approximately 25° (Fig 4B). Reconstructed movies of images captured serially in time showed a dynamic reorientation of the mitotic spindle relative to the position of the vacuole (Fig 4A; see S4 Video). Quantitative analysis of still images taken from Caco2^MYO5B−/−^ cells that displayed increased spindle rotation dynamics (i.e., an α-angle of more than 25°; c.f., Fig 4B) revealed an increase in the distance (*d*) measured between the vacuole rim and the spindle pole (Fig 4C) during the course of metaphase (Fig 4D and 4E, see S5 Video), supporting that the orientation of the mitotic spindle changed relative to the position of the vacuole. Thus, the presence of the vacuole influenced mitotic spindle orientation.

In addition to the mitotic spindle tilting, the presence of a large vacuole was also correlated with an increased frequency of 2 other mitotic complications: (1) a modest increase (from approximately 3% to approximately 8% of WT and knockout cells, respectively) in metaphase arrest frequency (see S4A–S4C Fig and S6 Video; note that, in cells that were not arrested in metaphase during the time span of the experiment, the presence of the vacuole did not affect the average duration of metaphase [S2A and S2B Fig]) and (2) an increase (from approximately 5% to approximately 12% of WT and knockout cells, respectively) in chromosome segregation difficulties (i.e., chromosome lagging, chromosome bridging, or micronuclei; see S5 Fig). Finally, vacuoles were also observed in Caco2^MYO5B−/−^ cells during cytokinesis, but no statistically significant difference in cytokinesis index between Caco2^MYO5B−/−^ cells with or without a vacuole was observed (see S4D and S4E Fig).

Thus, the mitotic spindle tilting defect and the increased incidence of metaphase arrest and chromosome segregation difficulties—but not the delay in cytokinesis—as observed in *MYO5B*-depleted cells was strongly correlated to the presence of large vacuoles.

### Loss of *MYO5B* expression causes the formation of giant late endosomes

**Fig 5:**
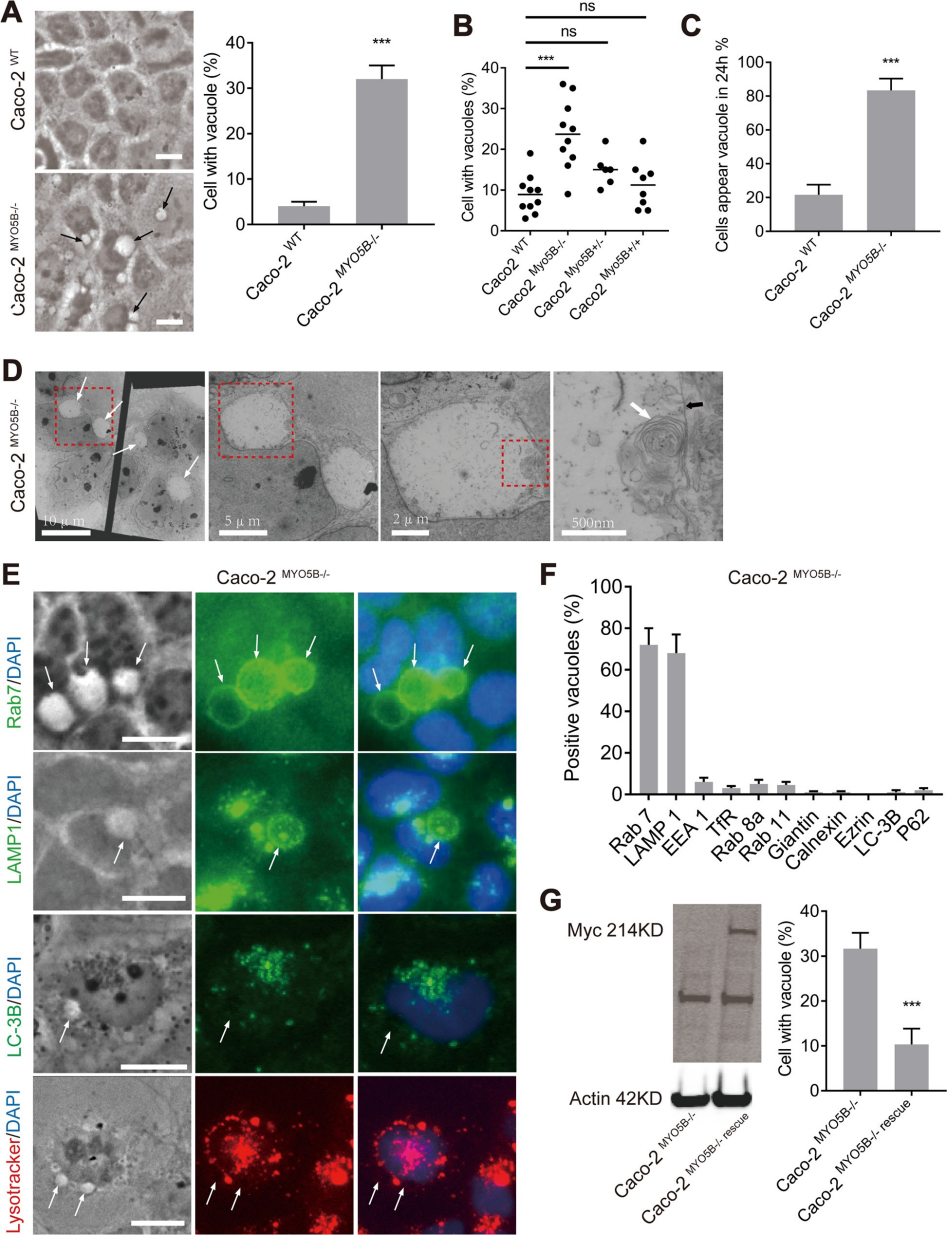
Loss of MYO5B expression causes the formation of giant late endosomes in Caco2^MYO5B−/−^ cells. (A) Arrows indicate large vacuoles in Caco2^MYO5B−/−^ cells. The percentage of cells with vacuole (diameter Ø > 1 μm) was quantified in fixed Caco2^WT^ and Caco2^MYO5B−/−^ cells. (B) Multiple Caco2^MYO5B−/−^ clones showed a higher percentage of cells with vacuoles compared with heterozygous or control clones. (C) The percentage of cells formed a vacuole (Ø > 1 μm) for at least 30 min during the 24 h was quantified in live cell imaging experiments. (D) Electron microscopy images of Caco2^MYO5B−/−^ cells revealed the large electron-lucent vacuoles (arrows) were with single membrane (black thick arrow) and presence of intraluminal material (white thick arrow). (E–F) Vacuoles (arrows) in Caco2^MYO5B−/−^ cells were positive for Rab7 and LAMP1 but virtually negative for lysotracker, LC-3B, and other various organelle markers. For using lysotracker, Caco2^MYO5B−/−^ cells were incubated in medium with lysotracker for 1 h before fixation. (G) Western blot showing myosin Vb expression after the re-expression of a CRISPR-Cas9–resistant human MYO5B cDNA in Caco2^MYO5B−/−^ cells (Caco2^MYO5B−/−^ rescue cells), and the ameliorating effect on the number of vacuoles. (A, C, F and G) *N* = 3 independent experiments. *t* test, ***p < 0.001. Error bars indicate + SD (bar graph). (A and E) Scale bars: 10 μm. Values for each data point in panels A, B, C, F, and G can be found in S5 Data. EEA, early endosomal antigen-1; KD, knockdown; LC, light chain; ns, not significant; WT, wild type.

We then focused on the identity of the vacuoles. We found that Caco2^MYO5B−/−^ cells, as such, displayed significantly more large vacuoles in the cytoplasm when compared with Caco2^WT^ cells (Fig 5A), and this was observed in multiple *MYO5B* knockout clones (Fig 5B). Large vacuoles were observed in approximately 30% of all fixed cells (Fig 5A). Live cell imaging, however, revealed that <20% of the Caco2^WT^ but >80% of the Caco2^MYO5B−/−^ cells formed a vacuole (Ø > 1 μm) for at least 30 min during the 24 h time course of the experiments (Fig 5C). Electron microscopy analysis confirmed the presence of large electron-lucent vacuoles in Caco2^MYO5B−/−^ cells (Fig 5D), and the presence of intraluminal material suggested resemblance to degradative compartments. Immunolabeling with antibodies against various organelle markers showed that the vacuoles were specifically positive for late endosome markers (i.e., late endosome–associated membrane protein [LAMP]1 and the small GTPase rab7) (Fig 5E and 5F; see S6 Fig and S7 Fig). No lysotracker or autophagosome markers, i.e., microtubule-associated protein 1 light chain (LC)-3B (Fig 5E and 5F) and sequestosome-1 (p62; see S6 Fig), were observed at the vacuoles, indicating that the vacuoles represented nonacidic and nonautophagic giant late endosomes. We also observed the presence of vacuolated LAMP1-positive late endosomes in villus enterocytes (but not in crypts) of the small intestine of *Myo5b* knockout mice and MVID patients carrying bi-allelic *MYO5B* mutations (see S8A–S8C Fig). Importantly, upon the re-introduction of myc-tagged full-length human myosin Vb in Caco2^MYO5B−/−^ cells, the vacuoles no longer formed (Fig 5G), demonstrating that the formation of the large vacuoles was causally related to the loss of *MYO5B* expression.

We also generated *MYO5B* knockout human embryonic kidney (HEK)293 cells (HEK293^MYO5B−/−^; see S9A Fig). When compared to WT HEK293 cells, HEK293^MYO5B−/−^ developed large vacuoles (S9B Fig), displayed therewith correlated mitotic spindle tilting (S9C–S9G Fig) and cell delamination (S10A and S10B Fig). Further, HEK293^MYO5B−/−^ displayed vacuole-unrelated delayed cytokinesis (S10C–S10E Fig). These results indicate that the observed effects were not specific for Caco2 cells.

**Fig 6:**
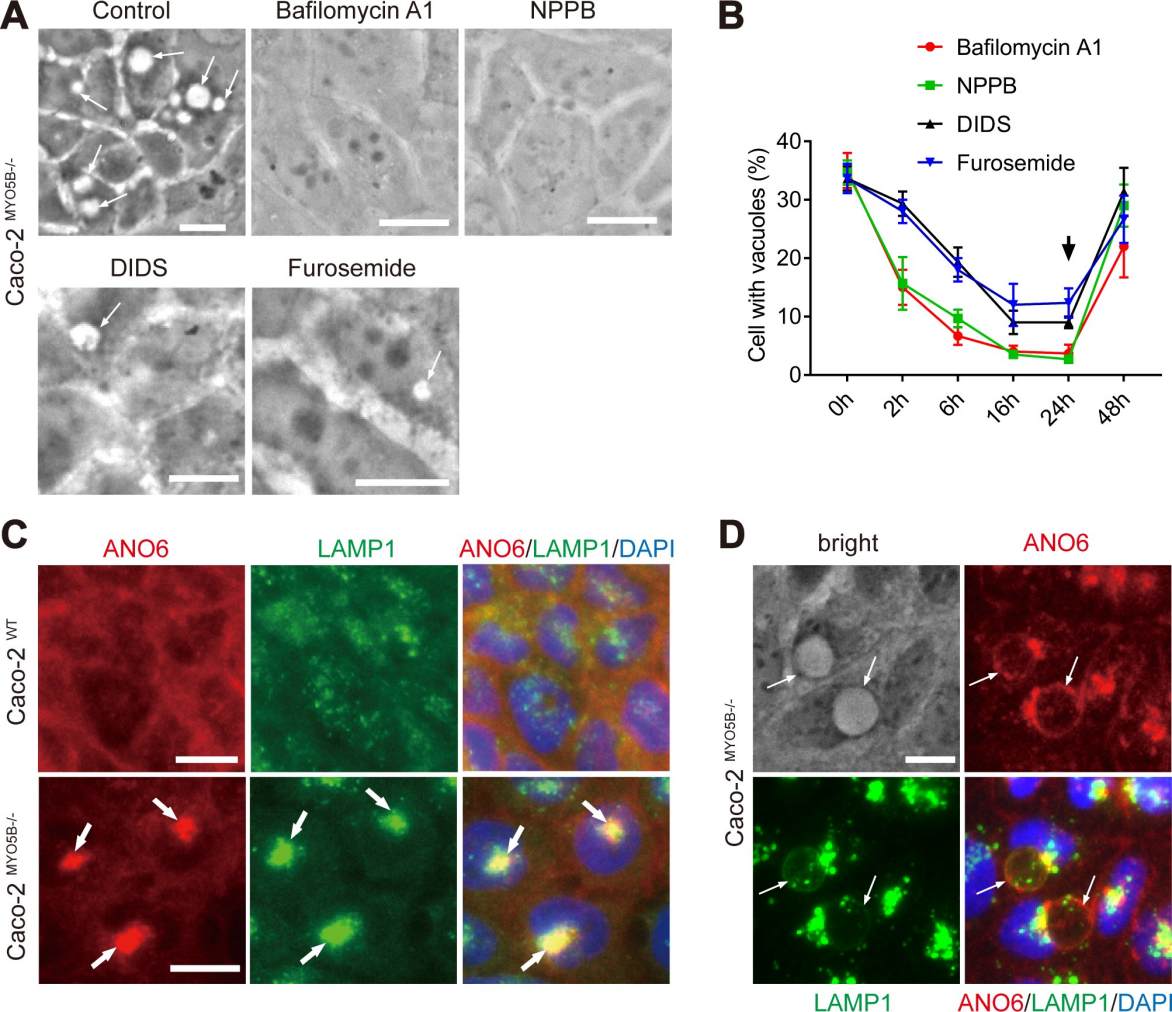
vATPase and chloride channel activities mediate the late endosomal phenotype in Caco2^MYO5B−/−^ cells. (A–B) The numbers of vacuoles in Caco2^*MYO5B*–/–^cells were reduced after treatments with vATPase inhibitor BafA1 and chloride channel inhibitors NPPB, DIDS, and furosemide for 24 h (A) and other appropriate times (B). Vacuoles reappeared after washout of the chemicals and culture in normal cell culture medium (B, arrow). Values for each data point can be found in S6 Data. (C–D) Caco2^*MYO5B*−/−^ cells revealed a shift in the abundance of the chloride channel ANO6 from the cell periphery to LAMP1-positive compartments (C, thick arrows) and vacuoles (D, thin arrows) compared with Caco2^WT^ cells. *N* = 3 independent experiments. Scale bars: 10 μm. ANO, anoctamin; Baf, bafilomycin; DIDS, 4,4'-Diisothiocyano-2,2'-stilbenedisulfonic acid; LAMP, late endosome–associated membrane protein; NPPB, 5-nitro-2-(3-phenylpropyl-amino) benzoic acid; vATPase, vacuolar H^+^-adenosine triphosphatase; WT, wild type.

We reasoned that understanding the mechanism that underlie the formation of giant late endosomes may provide tools to interfere with their formation and, hence, allow us to further test the relationship between the presence of the vacuoles and the mitotic spindle orientation defects. The distended appearance of the late endosomes suggested an ion imbalance and resembled the swollen late endosomes as seen in cells infected with *Helicobacter pylori*. *H*. *pylori* is a bacterium that inserts a vacuolating toxin with chloride channel activity into the host cell’s endosomal system and in this way causes late endosome ion imbalance and resultant late endosome swelling [21–23]. Indeed, inhibition of the vacuolar H^+^-adenosine triphosphatase (vATPase) proton pump activity with bafilomycin A1 (BafA1) or inhibition of chloride channel activity with either of 3 unrelated compounds (i.e., furosemide, 4,4'-Diisothiocyano-2,2'-stilbenedisulfonic acid [DIDS] and 5-nitro-2-(3-phenylpropyl-amino) benzoic acid [NPPB]) effectively and reversibly abolished the appearance of giant late endosomes in Caco2^MYO5B−/−^ cells (Fig 6A and 6B). Possibly, late endosome ion imbalance in *MYO5B*-depleted cells is the result of inhibited plasma membrane recycling of various ion transporters and their resultant accumulation in late endosomes. Indeed, loss of *MYO5B* expression is known to inhibit plasma membrane protein recycling and result in the appearance of plasma membrane proteins in late endosomes. Furthermore, in support of this, we observed a redistribution of a chloride channel, anoctamin (ANO)-6 [24,25], from the cell periphery to (giant) late endosomes upon loss of *MYO5B* expression (Fig 6C and 6D).

**Fig 7:**
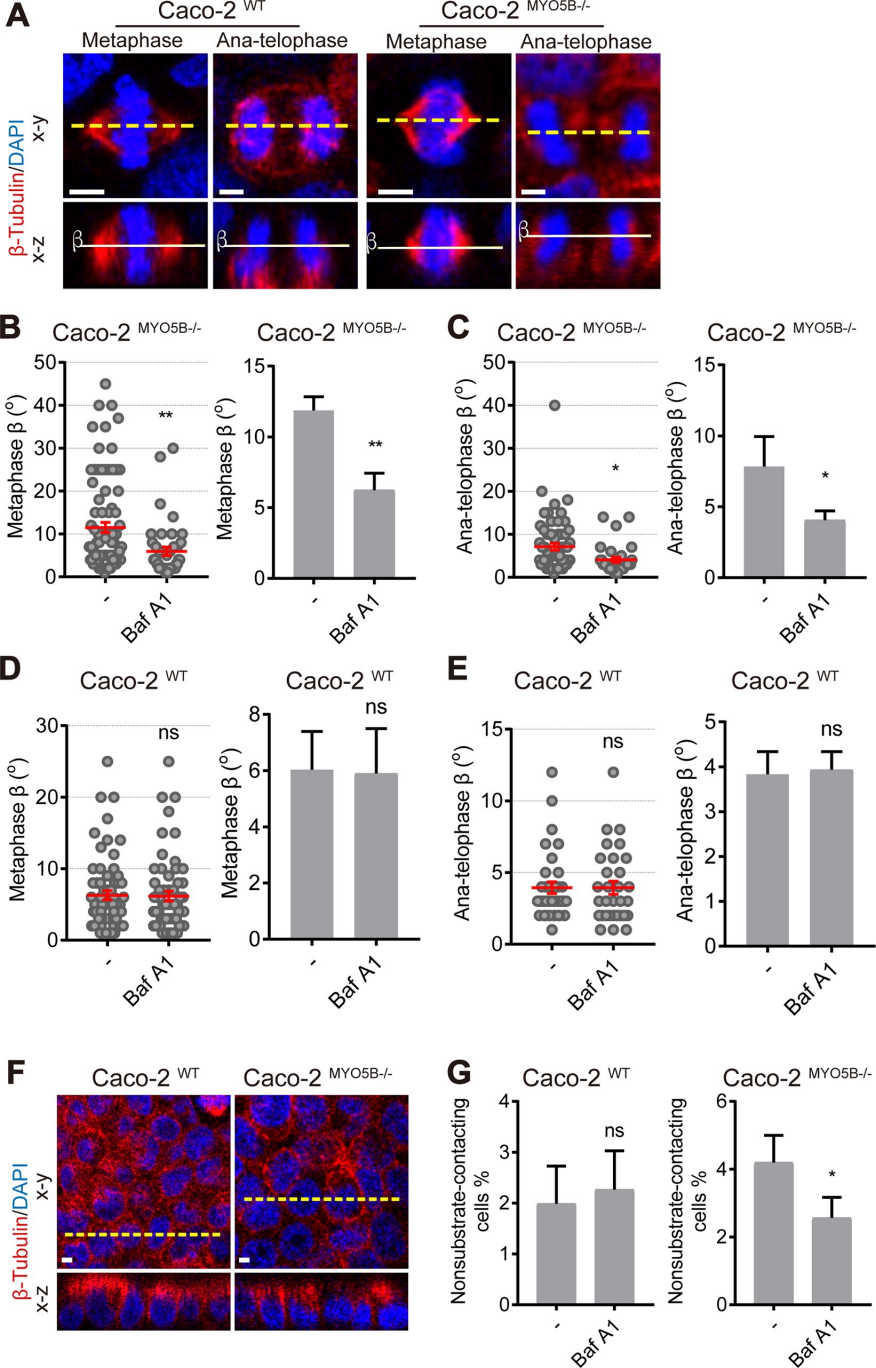
vATPase inhibitor Baf A1 rescues the spindle orientation defects in Caco2^MYO5B−/−^ cells. (A) Caco2^WT^ and Caco2^*MYO5B−/−*^ cells were treated with Baf A1 for 24 h before fixation and stained as indicated. The β-angle represents the angle between the spindle axis and the substratum in the confocal x-z dimension. (B–E) The β-angle in metaphase and anatelophase was quantified in Caco2^WT^ and Caco2^*MYO5B−/−*^ cells after treated with Baf A1. The dot graph: each dot indicates one cell’s β-angle. The histogram graph: the statistical analysis of the mean for each experiment. *n* ≥ 9 cells/experiment were analyzed for *N* = 3 independent experiments. (B) Caco2^*MYO5B−/−*^ in metaphase, (C) Caco2^*MYO5B−/−*^ in anatelophase, (D) Caco2^WT^ in metaphase, (E) Caco2^WT^ in anatelophase, (F, G) The percentage of nonsubstrate-contacting cells was quantified in Caco2^WT^ and Caco2^*MYO5B−/−*^ cells after treated with Baf A1. *N* = 3 independent experiments. *t* test, *p < 0.05,**p < 0.01. Error bars indicate ± SEM (dot graph) or + SD (bar graph). Scale bars: 5 μm. Values for each data point in panels B, C, D, E and G can be found in S7 Data. baf,; ns, not significant; WT, wild type.

Notably, treatment of Caco2^MYO5B−/−^ cells with BafA1 not only rescued the giant late endosome phenotype but also normalized the tilted spindle orientation to control values (Fig 7A–7C). Treatment of WT cells with BafA1 did not affect spindle orientation (Fig 7D and 7E). Further treatment of Caco2^MYO5B−/−^ cells with BafA1 abolished cell delamination (Fig 7F and 7G). These results support the relationship between the presence of the vacuoles and the mitotic spindle orientation defects.

### Rab7 availability controls the formation of giant late endosomes and mitotic spindle orientation defects in *MYO5B*-depleted cells

**Fig 8:**
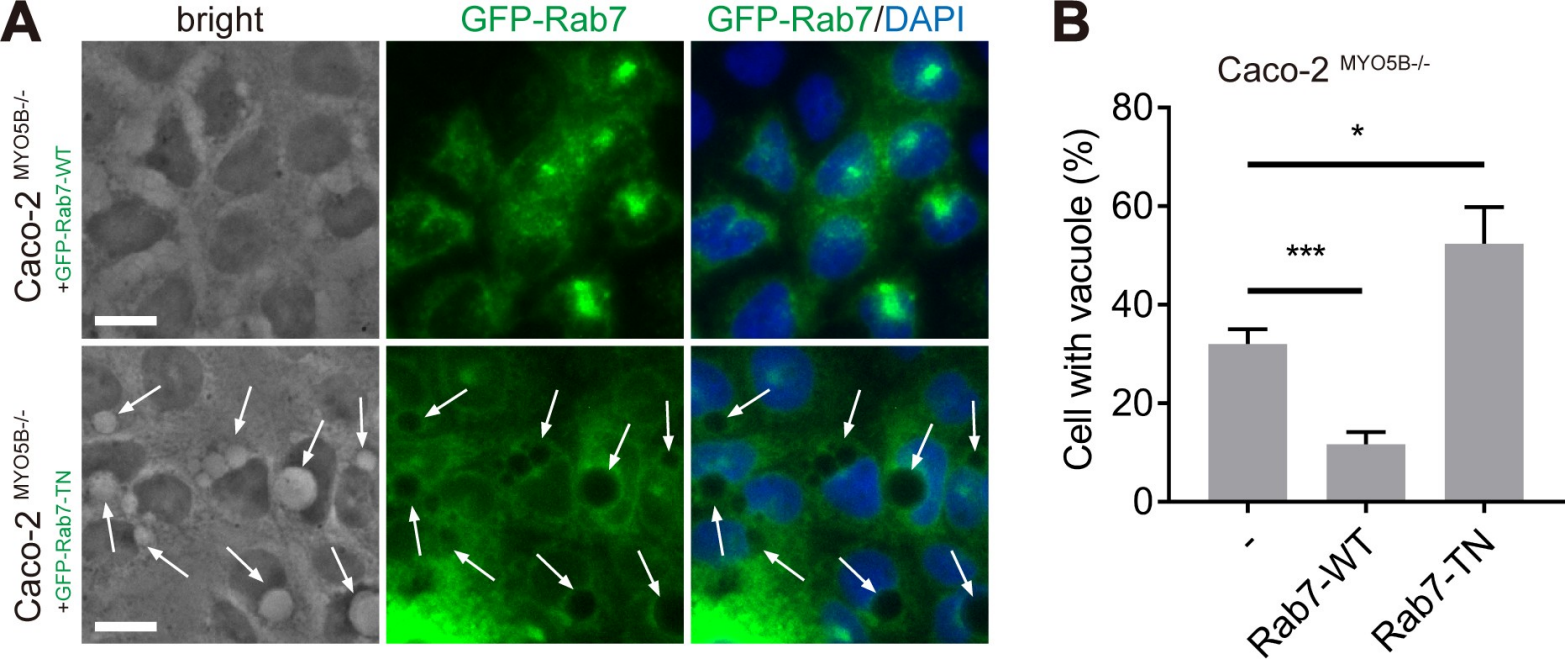
Rab7 controls the formation of giant endosomes. (A, B) Quantification of the vacuoles (A, arrows) percentage of Caco2^*MYO5B*–/–^cells expressing GFP-rab7-WT or GFP-rab7-T22N. *N* = 3 independent experiments. Values for each data point can be found in S8 Data. Student *t* test, *p < 0.05, ***p < 0.001. Error bars indicate + SD. Scale bars: 10 μm. GFP, green fluorescent protein; WT, wild type.

Rab7 is a key regulator of late endosome dynamics [26] and has been reported to correct trafficking defects in lysosomal storage disease when overexpressed [27]. Our data demonstrate that the overexpression of an enhanced green fluorescent protein (eGFP)-tagged WT(rab7-WT) or eGFP-tagged dominant-negative (DN) rab7 mutant (rab7-T22N) in Caco2^MYO5B−/−^ cells inhibited or exaggerated the formation of giant late endosomes, respectively (Fig 8A and 8B).

**Fig 9:**
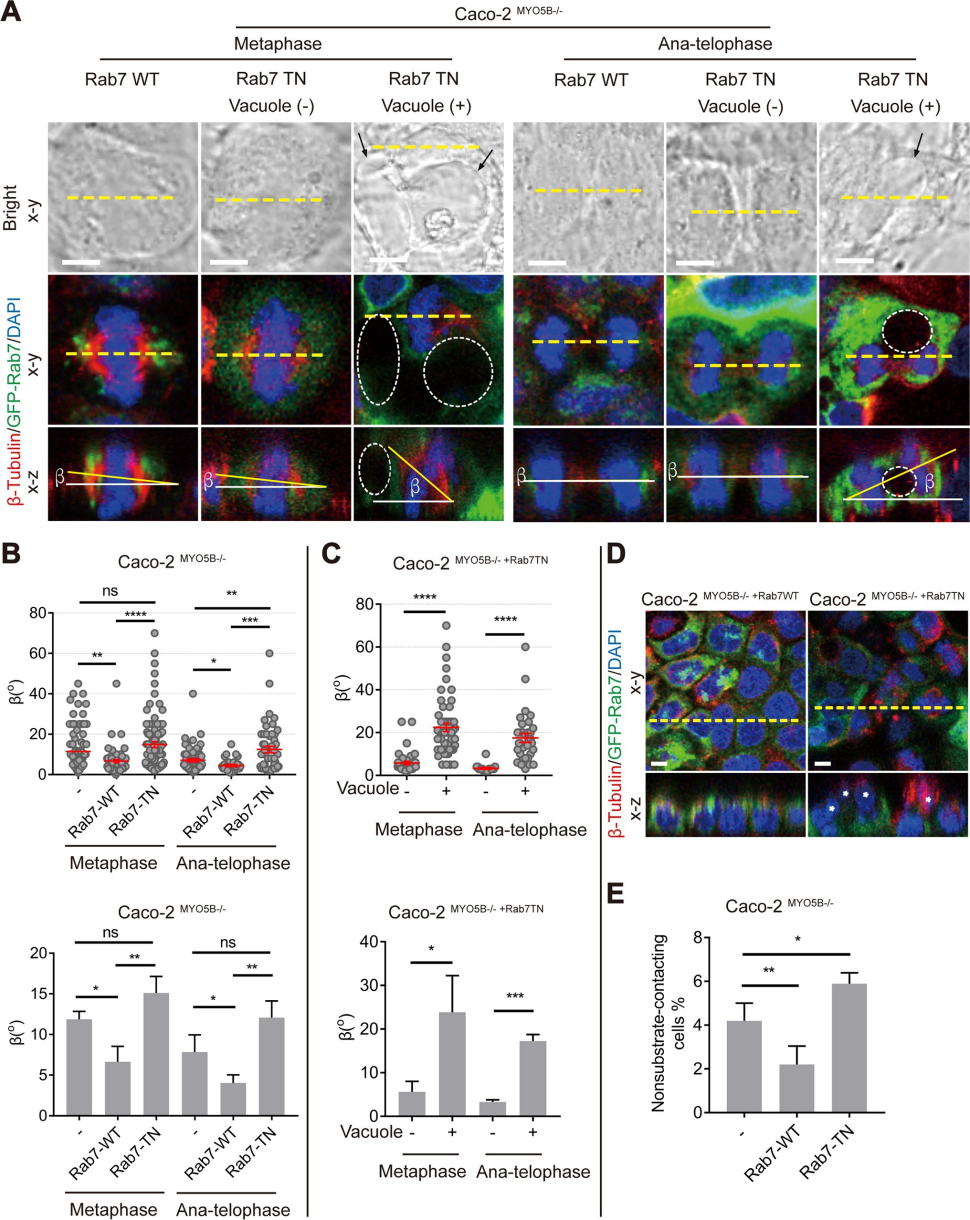
Rab7 controls the formation of giant endosomes and mitotic spindle orientation defects in Caco2^MYO5B−/−^ cells. (A) Caco2^*MYO5B−/−*^ cells expressing GFP-rab7-WT or GFP-rab7-T22N in metaphase and anatelophase were fixed and stained as indicated. Arrows in bright field and dotted circles in fluorescent field indicate large vacuoles. (B) The β-angle was quantified in Caco2^*MYO5B−/−*^ cells expressing GFP-rab7-WT or GFP-rab7-T22N during metaphase and anatelophase. (Upper graph) Each dot indicates one cell’s β-angle. (Bottom graph) The statistical analysis of the mean for each experiment. *n* ≥ 6 cells/experiment were analyzed for *N* = 3 independent experiments. (C) The quantification of β-angle in Caco2^*MYO5B−/−*^ cells expressing GFP-rab7-T22N with and without vacuoles. (Upper graph) Each dot indicates one cell’s β-angle. (Bottom graph) The statistical analysis of the mean for each experiment. *n* ≥ 6 cells/experiment were analyzed for *N* = 3 independent experiments. (D–E) The percentage of cells not contacting the substratum (D, asterisks) was quantified in Caco2^*MYO5B−/−*^ cells expressing GFP-rab7-WT or GFP-rab7-T22N. *N* = 3 independent experiments. *t* test, *p < 0.05, **p < 0.01, ***p < 0.001, ****p < 0.0001. Error bars indicate ± SEM (dot graph) or + SD (bar graph). Scale bars: 5 μm. Values for each data point in panels B, C, and E can be found in S9 Data. GFP, green fluorescent protein; ns, not significant; WT, wild type.

We took advantage of the modifying effects of rab7 to further address the causality between the presence of giant late endosomes and mitotic spindle orientation defects upon the loss of *MYO5B* expression. We found that the overexpression of rab7-WT, but not of the inactive rab7-T22N mutant, in Caco2^MYO5B−/−^ cells not only effectively prevented the formation of giant late endosomes but also normalized the mitotic spindle tilting range relative to the substratum (β-angle) in metaphase and anaphase cells (Fig 9A–9C). Furthermore, the overexpression of rab7-WT, but not of rab7-T22N, prevented the increase in Caco2^MYO5B−/−^ cell delamination (Fig 9D and 9E). The expression of rab7-WT or rab7-T22N in Caco2^MYO5B−/−^ cells did not affect the observed delay in cytokinesis (see S11 Fig), which is in agreement with the nonsignificant correlation between the presence of giant late endosomes and cytokinesis duration. Together these results indicate a causal relationship between the presence of giant late endosomes, spindle orientation defects, and epithelial cell delamination, but not cytokinesis delay, in cells that have lost *MYO5B* expression.

### Wortmannin-induced giant endosomes correlate with mitotic spindle orientation defects

**Fig 10:**
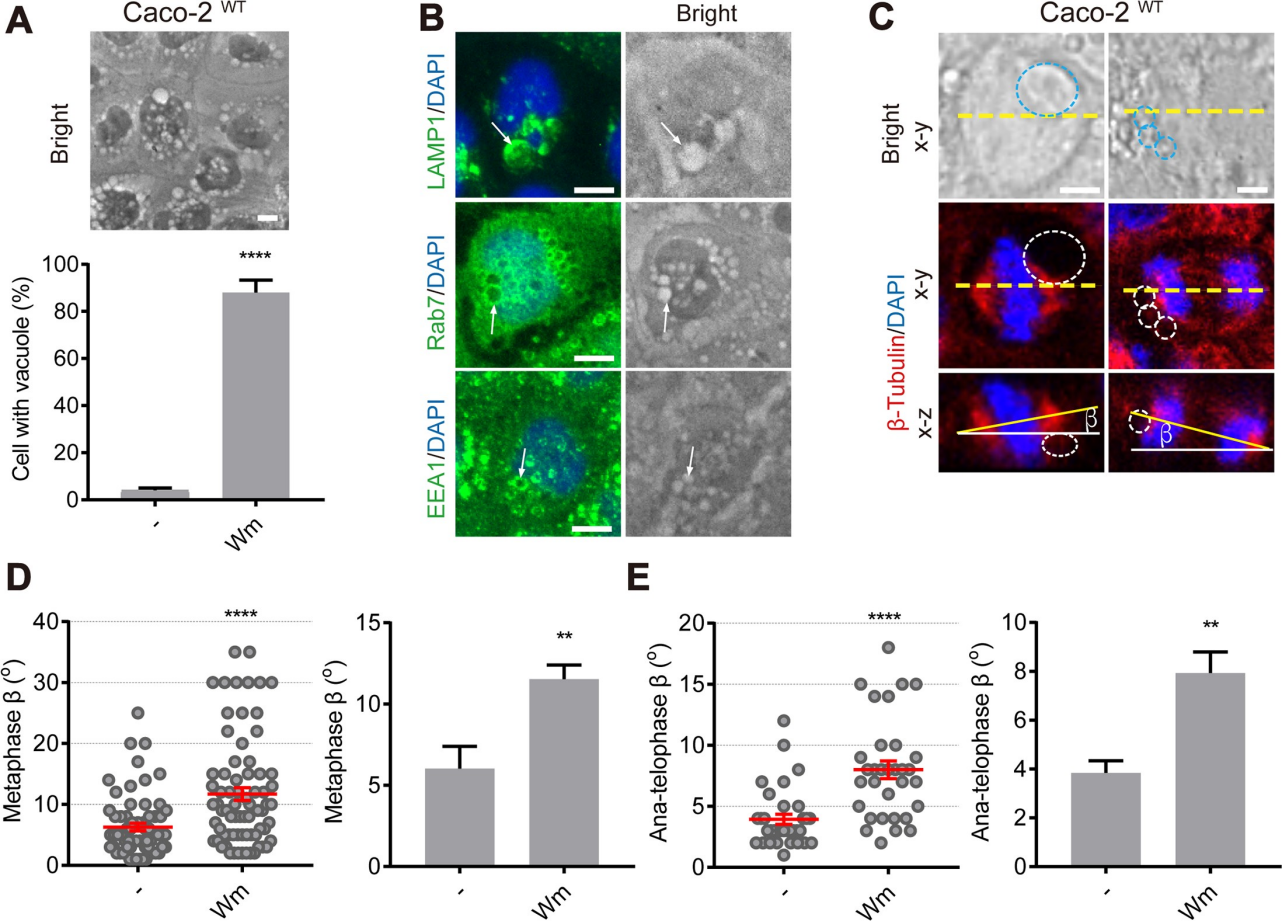
PI3K inhibitor Wm induces large vacuoles and mitotic spindle orientation defects in Caco2^WT^ cells. (A) The numbers of Caco2^WT^ cells with vacuoles (diameter Ø > 1 μm) are increased after treated with Wm for 1 h. (B) The vacuoles (arrows) induced by Wm are positive for LAMP1, Rab7 and EEA1. (C) Caco2^WT^ cells treated with Wm were fixed and stained as indicated. The dotted circles in the images indicate large vacuoles during metaphase and anatelophase. (D, E) The β-angle in metaphase (D) and anatelophase (E) was quantified in Caco2^WT^ cells after being treated with Wm. The dot graph: each dot indicates one cell’s β-angle. The histogram graph: the statistical analysis of the mean for each experiment. More than 9 cells/experiment were analyzed for N = 3 independent experiments. *t* test, **p < 0.01, ****p < 0.0001. Error bars indicate ± SEM (dot graph) or + SD (bar graph). Scale bars: 5 μm. Values for each data point in panels A, D, and E can be found in S10 Data. EEA, early endosomal atigen-1; LAMP, late endosome–associated membrane protein; Wm, Wm; WT, wild type.

We reasoned that if giant endosomes, rather than the mere loss of *MYO5B*, was responsible for the mitotic spindle orientation phenotype, the induction of giant endosomes via a different mechanism should also result in the spindle orientation phenotype. To address this issue, we treated WT Caco2 cells with wortmannin (Wm), a fungal metabolite and potent inhibitor of phosphatidylinositol-3 kinases that was previously reported to induce endosomal vacuoles in different cell types [28–30]. Treatment of WT Caco2 cells with Wm induced the formation of large endosomal vacuoles with comparable sizes as seen in *MYO5B*-depleted cells (Fig 10A). Antibody labeling showed that these vacuoles were positive for markers of both early (EEA1) and late (rab7, LAMP1) endosomes (Fig 10B). Wm-treated WT Caco2 cells that formed large vacuoles displayed a mitotic spindle tilting phenotype similar to that seen in vacuole-containing *MYO5B*-depleted cells (Fig 10C–10E). These data thus support a physical occlusion model in which the presence of giant endosomes, rather than other myosin Vb-mediated processes, caused tilting of the mitotic spindle apparatus.

## Discussion

In this study, we demonstrate that the presence of giant late endosomes in the cytoplasm of mitotic epithelial cells can influence the orientation of the mitotic spindle apparatus, lead to the misorientation of the plane of cell division during the course of mitosis, and increase the delamination of epithelial cells. This is the first evidence that aberrant endosome size, presumably through physically hindering the alignment of the mitotic spindle with the substratum, can derange cell division orientation and epithelial monolayer organization and, hence, underscores the need for proliferative epithelial cells to restrict and maintain (late) endosome size when entering mitosis.

Large organelles such as the Golgi apparatus are typically fragmented into smaller units prior to mitotic entry, and the inability of cells to fragment their Golgi prevents mitotic entry [31]. Endosomes in mammalian cells are relatively small with typical narrow size distributions and do not show fragmentation during mitosis [32,33]. Except perhaps for the observed metaphase arrest in some cells, the presence of giant late endosomes did not robustly trigger a mitotic checkpoint. This suggests that cells do not actively monitor endosome size in mitosis and, therefore, that the mitotic process is vulnerable to deregulated endosome size.

Giant late endosomes developed as a direct result of loss of *MYO5B* expression, which was supported in vivo by the presence of aberrantly sized late endosomes in intestinal epithelial cells in *Myo5b* knockout mice and MVID patients with *MYO5B* mutations. Disorganized LAMP1-positive compartments were previously also reported in *Myo5b* knockout (KO) mice by Engevik and colleagues [34] and, using electron microscopy, Vogel and colleagues reported numerous large lysosomes among other vesicular-tubular compartments in MVID patients’ enterocytes [35]. *MYO5B* encodes the recycling endosome–associated myosin Vb protein which, when mutated in MVID patients, causes in villus epithelial cells the redistribution of cell surface proteins to intracellular vesicles among which are late endosomes [4,36,37]. The formation of giant late endosomes in *MYO5B*-depleted cells was dependent on proton pump and chloride channel activity. Together with their vacuolated morphology at the ultrastructural level and the observed redistribution of chloride channels to late endosomes in *MYO5B*-depleted cells, it is conceivable that the giant late endosomes formed as a result of perturbed late endosomal ion balances and resultant swelling. Thus, loss of *MYO5B* expression not only affects plasma membrane homeostasis but also late endosomal homeostasis and size. It remains to be determined which exact cargoes are functionally important for the late endosome phenotype and for the mitotic spindle orientation defect. It is possible that many cargoes with small but additive effects are responsible.

Our results are particularly significant because previous studies have shown that recycling endosomes and therewith associated rab11a contributed to the regulation of mitotic spindle positioning and orientation [12,13]. The mitotic defects resulting from the loss of rab11a [12] or *MYO5B* (this study) appear distinct at the mechanistic level because loss of rab11a did not induce giant late endosomes [38–40] as observed here in *MYO5B*-depleted cells, and loss of *MYO5B* did not cause metaphase delay or affect astral microtubules (this study) as reported in rab11a-inhibited cells [12]. Moreover, spindle orientation defects in *MYO5B*-depleted cells could be mimicked fully rescued by overexpression of the late endosome–associated rab7. The mechanisms via which rab7 achieves this is not clear, but the finding further supports the requirement of late endosomes to induce the spindle orientation defects upon loss of *MYO5B* expression. Our in vivo data from *Myo5b* knockout mice, which developed late endosome vacuoles in their villus cells but not in their proliferative crypt cells, indicate that the mere loss of *Myo5b*, i.e., without vacuoles present, is not sufficient to induce a spindle orientation defect. In agreement, the observation that the induction of endosomal vacuoles in WT cells by Wm similarly resulted in tilted mitotic spindles and cell delamination support a model in which it was the presence of giant endosomes, rather than other myosin Vb-mediated processes, that caused tilting of the mitotic spindle apparatus Together, the data presented in this study thus identify deregulated late endosomal size as another mechanism that can contribute to aberrant mitotic spindle orientation in response to the loss of a recycling endosome–associated protein. These findings add an unexpected new dimension to the role of membrane trafficking proteins in processes that ensure proper mitotic progression and cell division.

It should be noted that although virtually all mitotic cells that showed increased tilting of their spindle apparatus contained a giant late endosome, not all mitotic cells with a giant late endosome showed increased tilting of their spindle apparatus and multilayering. This apparent discrepancy, i.e., that not all cells with a giant late endosome showed such mitotic phenotype, may be attributed to (i) the size and position of the vacuole relative to the spindle pole, concomitant with (ii) its position relative to the spindle pole in the x-z direction (lateral positioning affects spindle orientation in the plane (x-y) of the monolayer), (iii) the existence of a recently reported spindle tilting correction mechanism during anaphase [41], and (iv) the reintegration of delaminated cells in the monolayer.

Recycling endosomes and therewith associated rab11a have also been shown to contribute to cytokinesis [10]. In addition to mitotic spindle misorientation, a delay in cytokinesis was observed upon the loss of *MYO5B* expression. Previous studies have demonstrated that rab11a-positive recycling endosomes, via the rab11-binding partner FIP3 [42], mediate the delivery of the actin depolymerizing p50RhoGAP [43] and other proteins [44] to the ingressing cleavage furrow during late telophase to promote further ingression and abscission. Also, motor proteins have been implicated in the delivery of recycling endosomes to the cleavage furrow [11,45,46]. Our results demonstrate a role for myosin Vb in cytokinesis, and future studies are needed to unravel the mechanism. Notably, in contrast to the mitotic spindle misorientation, the delay in cytokinesis did not correlate with the presence of giant late endosomes and could not be rescued by the overexpression of rab7, arguing against a role for the giant late endosomes, and possibly in favor of a functional relationship with rab11a, in the cytokinesis phenotype.

We consider it unlikely that the giant late endosome-induced spindle orientation phenotype contributes to the pathogenesis of MVID because the giant late endosomes present in MVID intestine predominantly appear in differentiated villus enterocytes and not in proliferating crypt cells. It should be noted that our results were obtained primarily in cell culture which does not fully recapitulate intestinal physiology. The intestine of the *Myo5b* knockout mouse may show adaptive physiological responses that involve proliferation of crypt cells, and in vivo conditions and outcomes may therefore be different. The physiological relevance of the mechanisms described here in the in vitro systems thus remains to be elucidated. Future studies should address the possible contribution of oversized endosome-related spindle misorientation to the pathogenesis of human diseases in which aberrantly sized endosomes have been reported [47], which include neurodegenerative diseases [48,49], infectious diseases [21], rare inherited diseases [50–53], and cancer [54,55], and the potential of modulating rab7 availability as a therapeutic avenue.

## Materials and methods

### Ethics statement

All animal experiments comply with the Guidelines of the European Union Council (2010/63/UE) and of the Spanish Government (RD 53/2013) and were approved by the Ethical committee of Animal Experimentation at Vall d’Hebron Institute of Research (80/12 CEEA).

### Cells and tissues

Human Caco2 cells (HTB-37) and HEK293 cells (CRL-1573) (ATCC, Gaithersburg, MD, USA)were grown in DMEM (Thermo Scientific Fisher, Waltham, MA, USA), supplemented with 10% heat-inactivated fetal calf serum (Invitrogen) and antibiotics (penicillin, streptomycin) (Thermo Scientific Fisher, Waltham, MA, USA), and maintained at 37°C in a humidified atmosphere with 5% CO_2_. For experiments, cells were typically cultured in Lab-Tek chambers (Thermo Scientific, Waltham, MA, USA) or on Transwell semipermeable polycarbonate filter supports (Corning, New York, NY, USA3401). Tissues from MVID patients and age-matched control were described by Szperl and colleagues, Golachawska and colleagues, and Dhekne and colleagues [56–58].

### Reagents and plasmids

Commercial antibodies used for immunofluorescence and Western blot are listed in S1 Table. The plenti-plasmids Rab7-WT-GFP and Rab7-GFP-T22N DN mutant were gifts from Peter van der Sluijs (University Medical Center Utrecht, the Netherlands). The plenti-plasmids β-Tubulin-GFP (64060) and Histone2B-mCherry (51007) are from Addgene (Watertown, MA, USA).

### Virus production and transduction

Lentiviral particles were created using a second-generation system. Briefly, following the instruction of Fugene (Promega, Madison, WI, USA), the mixture of pLenti-plasmid, vesicular stomatitis virus-glycoprotein, delta-8.91, and Fugene was added in the 6-well plate, and 1 × 10^6^ HEK293T cells were seeded on top of the mixture. After overnight incubation, medium was changed with DMEM with 2.5% (v/v) serum and 1% antibiotics. After 48 h, viral particles were harvested and filtered through 0.45-μm filter (GE healthcare, Hatfield, UK). Cells were transduced by filtered virus supernatant with polybrene (8ug/ml) 6 h after seeding. The next day virus medium was changed with selection medium containing appropriate antibiotics.

### Generation of cell lines

*MYO5B* knockout cells were generated using the CRISPR/Cas9 genome editing system. DNA Oligos were the following: sense: caccgGATATCTGGATTCCGTAAGA; antisense: aaacTCTTACGGAATCCAGATATCc.

Virus production and cell transduction procedures were as described above. Transduced cells were selected with puromycin (5 μg/ml, Sigma-Aldrich, St. Louis, MO, USA) 1 day after infection. After 3 days, infected cells were sparsely plated, and individual clones were picked. Finally, the presence of a premature stop codon was checked by sequencing (GATC Company), and myosin Vb expression levels were checked by western blot. Caco2 cells expressing Rab7-WT-GFP or Rab7-GFP-T22N, β-Tubulin-GFP, and Histone2B-mCherry were generated by virus transduction with plenti-plasmids as described above.

### *MYO5B* rescue assay

CRISPR-cas9–resistant *MYO5B* cDNA was generated by site-directed mutagenesis (NEB, Hitchin, UK), introducing a silent C>G mutation in the pENTR1a-*MYO5B* corresponding to the 60th amino acid in the WT myosin Vb protein. The primers used to mutate *MYO5B* cDNA were: forward–ACCAGCTGCC**G**TTCTTACGGA-, reverse–TGCGTTGTACATCAATTGGG-. The myc-tagged CRISPR-cas9–resistant *MYO5B*cDNA was transferred to pLenti-plasmid with blasticidin-resistance. Caco2^*MYO5B−/−*^ cells were transduced with virus containing the CRISPR-cas9−resistant *MYO5B* cDNA. After blasticidin (6 ug/ml, Thermo Scientific, Waltham, MA, USA) selection, the expression of the myc-tag was checked by western blotting and immunofluorescence.

### *Myo5b* intestinal knockout mice

*Myo5b*tm1c conditional *Myo5b* knockout mice on a C57BL/6 background were generated by crossing *Myo5b*tm1a(KOMP)Wtsi mice [59] with C57BL/6 Tg(CAG-Flpo)1Afst (Flp delete; EMMA ID EM:05149) [60] animals. To obtain intestinal-specific *Myo5b*^−/−^ mice, *Myo5b*tm1c animals were crossed with a C57BL/6 Tg(Vil-cre/ERT2)23Syr mice (Vil-CreERT2; The Jackson Laboratory Stock No: 020282) [61], carrying a tamoxifen inducible Cre recombinase expressed under the control of the Villin promoter. *Myo5b* deletion was achieved by a single i.p. injection of 4 mg tamoxifen (Sigma-Aldrich, St. Louis, MO, USA), and animals were killed by cervical dislocation on day 5 after Cre induction. The orientation of the mitotic spindle in small intestinal cells was determined in hematoxylin and eosin stained sections of WT and iKO mice by measuring the angle formed by mitotic metaphase/anaphase cells and the epithelial surface. At least 70 mitosis were scored in 4 female 10-week-old mice per group.

### Western blotting

Cells were harvested with lysis buffer (100 mM NaCl, 20 mM Tris-HCl [pH 7.6]), Triton X-100 and protease inhibitors (Roche, Almere, the Netherlands). Protein concentration was determined using the BCA protein assay (Sigma-Aldrich, St. Louis, MO, USA). Protein extracts (20 μg) were resolved on an SDS/4-15% polyacrylamide gel, transferred to nitrocellulose membranes, and blocked with Odyssey Blocking Buffer (LI-COR, Lincoln, NE, USA) at room temperature for 1 h. The membrane was incubated with primary antibodies (see S1 Table) at 4°C for 16 h, washed 3 times with PBS with 0.1% (v/v) Tween, and incubated with the secondary antibody in OBB/PBS buffer at room temperature for 1 h. Following washes with PBS with 0.1% (v/v) Tween, antibody signal was detected using Odyssey Imaging System (LI-COR, Lincoln, NE, USA).

### Immunofluorescence and confocal microscopy

For immunofluorescence microscopy, cells were fixed in 4% paraformaldehyde for 20 min and permeabilized with 0.2% (v/v) Triton X-100 at room temperature for 10 min. Cells were washed with PBS at each step. Cells were incubated in PBS with 1% (w/v) BSA at 37°C for 1 h, followed by overnight incubation with primary antibodies (see S1 Table) in PBS with 1% (w/v) BSA at 4°C. After washes with PBS, cells were incubated with secondary antibodies in PBS with 1% BSA (w/v) at room temperature for 1 h. Following washes with PBS, cells were mounted in Dako mounting medium and imaged using a fluorescence microscope (Leica, Frankfurt, Germany). In mitotic tilted experiments, cells were cultured in an 8-chambers plate (Lab-Tek II, 155409) and incubated with DMEM medium for 5 days. Cells were stained as above and imaged by TCS SP8 confocal microscope (Leica DMI 6000) (Leica, Frankfurt, Germany) with x-y-z stage.

### Electron microscopy

Tissues were fixed in 2% glutaraldehyde in 0.1 M sodiumcacodylate buffer at 4°C for 24 h. Cells grown in 6-well plates were fixed by adding dropwise an equal volume fixative (2% [v/v] glutaraldehyde, 2% [v/v] paraformaldehyde in 0.1 M sodiumcacodylate buffer). After 10 min, this mixture was replaced by pure fixative at room temperature for 30 min. After postfixation in 1% osmium tetroxide/1.5% potassium ferrocyanide (v/v, 4°C, and 30 min), tissues and cells were dehydrated using ethanol and embedded in EPON epoxy resin. A total of 60 nm sections were cut and contrasted using 2% (v/v) uranyl acetate in water followed by Reynolds lead citrate. Images were taken with a Zeiss Supra 55 in STEM-mode at 26KV using an external scan generator (Fibics, Canada) yielding mosaics of large area scans at 2.5 nm pixel resolutions.

### Immunohistochemistry

Immunohistochemistry was performed on 4 μm-thick tissue sections. The sections were deparaffinized, rehydrated in xylene and graded ethanol solutions. Sodium citrate buffer (10 mM sodium citrate, 0.05% (v/v) Tween-20, [pH 6.0]) was used for antigen retrieval, and slides were incubated overnight with primary antibodies (see S1 Table), followed by incubation with secondary antibodies and detection with horseradish peroxidase and 3,3'-diaminobenzidine. Slides were counterstained with hematoxylin and covered with mounting medium (Dako, Santa Clara, CA, USA).

### Treatment cells with different inhibitors

In experiments with vATPase and chloride channel inhibitors, cells were seeded on the coverslips in a 24-well plate and incubated with DMEM medium for 72 h. The vATPase inhibitor Baf A1 (0.1 μM), chloride channel inhibitors NPPB (200 μM), DIDS (500 μM), and furosemide (2 mM; Sigma-Aldrich, St. Louis, MO, USA) were added into the medium. After incubated with chemicals for indicated times, cells were fixed with PFA, followed by mounting of the slides and microscopical examination. In experiments with the PI3K inhibitor Wm (Sigma-Aldrich, St. Louis, MO, USA), cells were cultured as above and incubated with Wm (500 nM) for 1 h before fixation. In mitotic tilting and delamination experiments, cells were cultured in an 8-chambers plate (Lab-Tek II, 155409) (Thermo Scientific, Waltham, MA, USA)and incubated with DMEM medium for 5 days. Cells were treated with Baf A1 (0.1 μM) for 24 h or Wm (500 nM) for 1 h before fixation. Then cells were immunolabeled as described above and imaged with a TCS SP8 confocal microscope (Leica DMI 6000) (Leica, Frakfurt, Germanywith x-y-z stage.

### Live cell imaging

Cells expressing β-tubulin-GFP and histone2B-mCherry were seeded in an 8-chambers plate (Lab-Tek II, 155409) (Thermo Scientific, Waltham, MA, USA) and incubated with DMEM medium for 72 h. Then the live cells were observed 24 h with the laser scanning confocal microscope (GE Healthcare Bio-Sciences, Marlborough, MA, USA) with temperature and CO_2_ control. Images were collected using a 40× oil objective with GFP-mCherry and DIC channels every 6 to 8 min. Images were processed by Imaris software (Oxford Instruments, Zurich, Switzerland; https://imaris.oxinst.com/downloads).

### Quantification and statistical analysis

For live cell imaging and mitotic tilted experiments, images were performed with confocal microscope with x-y-z stage. In other immunofluorescence experiments, images were 2D object with x-y stage. All experiments were performed for at least 3 independent experiments. The measurements of the α-angle in horizontal rotation experiment were obtained with the free ImageJ tools (https://imagej.nih.gov/ij/download.html) in x-y confocal sections. The line of dividing direction was drawn vertically to the chromosome plane through the center in metaphase and drawn through the both the 2 daughter cells’ chromosome plane in anatelophase. The β-angle was obtained with the ImageJ tools x-z confocal sections. The indicated vacuole’s position was confirmed by bright field images. To estimate the percentage of cells not contacting the substratum, 5 random x-y fields of 4 × 10^4^ μm^2^ were analyzed in the x-y-z dimensions for 3 independent experiments. For statistical estimation, we used GraphPad Prism version 7.0 software (Graphpad software, San Doego, CA, USA) (https://www.graphpad.com/scientific-software/prism). Graphs represent mean y ± SEM or + SD, as indicated, *n* represents the numbers of cells analyzed. Statistical significance was determined by unpaired 2-tailed *t* tests.

## Supporting information

S1 Fig Characterization of Caco-2^*MYO5B−/−*^ cells. (A) Western blotting analysis showed a complete loss of the myosin Vb protein in Caco-2^*MYO5B−/−*^ cells. (B) The sequence of Caco-2^*MYO5B−/−*^ cells showed a deletion in exon 3 resulting in premature stop codon at amino acid position 66.
(TIFF)

S2 Fig Caco-2^*MYO5B−/−*^ cells show mitotic spindle orientation defects in transwell culture. (A, B) The β-angle in metaphase (A) and anatelophase (B) was quantified in Caco2^WT^ cells and Caco-2^*MYO5B−/−*^ cells after cultured in transwell for 5 days. The dot graph: each dot indicates one cell’s β-angle. The histogram graph: the statistical analysis of the mean for each experiment. More than 10 cells/experiment were analyzed for *N* = 3 independent experiments. (C) The percentage of nonsubstrate-contacting cells was quantified in Caco2^WT^ and Caco2^*MYO5B−/−*^ cells after cultured in transwell for 5 days. *N* = 3 independent experiments. *t* test, *p < 0.05, **p < 0.01, ***p < 0.001. Error bars indicate ± SEM (dot graph) or + SD (bar graph). Scale bars: 5 μm. Values for each data point can be found in S11 Data. WT, wild type.
(TIFF)

S3 Fig Mitotic spindle orientation in small intestinal epithelial cells. (A–C) The orientation of the mitotic spindle of small intestinal epithelial cells from Myo5b WT (A) and Myo5b iKO (B) mice by measuring the angle (α) formed by the mitotic cell in metaphase or anaphase (dotted red line) and the epithelial surface (solid red line) in hematoxylin and eosin stained sections (C). (D) The percentage of mitotic cells with angles in the indicated intervals is shown for Myo5b WT and iKO mice. At least 70 mitosis were scored in 4 animals per group. Fisher’s exact test of <10° versus ≥ 10°, *p* = 0.11. Values for each data point can be found in S12 Data. iKO, intestinal knockout; WT, wild type.
(TIFF)

S4 Fig Live cell imaging of mitotic Caco-2^*MYO5B−/−*^ cells. (A) The quantification of metaphase duration time in live Caco-2^*MYO5B−/−*^ cells with vacuole and without vacuole. Left side graph: each dot indicates one cell’s metaphase duration time; right side graph: the statistical analysis of the mean for each experiment. *n* > 10 cells/experiment were analyzed for *N* = 3 independent experiments. (B) Live cell imaging shows x-y-t time-lapse mitosis images on Caco-2^*WT*^and Caco-2^*MYO5B−/−*^ cells expressing β-tubulin-GFP and histone2B-mCherry. The metaphase arrest was identified by metaphase duration time >5 h and no observed division at the end. (C) Left side graph: quantification of the percentage of metaphase arrest in live Caco-2^WT^ and Caco-2^*MYO5B−/−*^ cells. Right side graph: quantification of the percentage of metaphase arrests in live Caco-2^*MYO5B−/−*^ cells with and without vacuoles. (D) The percentage of cytokinesis was quantified in the fixed Caco-2^*MYO5B−/−*^ cells with or without vacuoles. (E) The time of cytokinesis duration was quantified in live Caco-2^*MYO5B−/−*^ cells with or without vacuoles. Left side graph: each dot indicates one cell’s cytokinesis duration time; right side graph: the statistical analysis of the mean for each experiment. n > 10 cells/experiment were analyzed for *N* = 3 independent experiments. *t* test, *p < 0.05, **p < 0.01. Error bars indicate ± SEM (dot graph) or + SD (bar graph). Scale bars: 2 μm. Values for each data point in panels A, C, D, and E can be found in S13 Data. ns, not significant; WT, wild type.
(TIFF)

S5 Fig Loss of *MYO5B* causes chromosomal segregation errors. (A) The images show chromosomal segregation errors in Caco-2^*MYO5B−/−*^ cells, including chromosomal lagging and bridge in anatelophase and micronuclei in metaphase (arrows). (B) The chromosomal segregation errors were quantified in Caco2^WT^ and Caco-2^*MYO5B−/−*^ cells. (C) The chromosomal segregation errors were quantified in Caco-2^*MYO5B−/−*^ cells with and without vacuoles. (D and E) The chromosomal segregation errors in Caco2^WT^ (D) and Caco-2^*MYO5B−/−*^ cells (E) were compared after treated with Baf A1 for 24 h. *N* = 3 independent experiments. *t* test, *p <0.05. Error bars indicate + SD. Scale bars: 5 μm. Values for each data point in panels B through E can be found in S14 Data. Baf, bafilomycin; ns, not significant; WT, wild type.
(TIFF)

S6 Fig Immunolabeling of Caco-2^*MYO5B−/−*^ cells. Vacuoles (asterisks) in Caco-2^*MYO5B*−/−^ were found to be negative for markers of organelles including Golgi (giantin), endoplasmic reticulum (calnexin), microvillus inclusion bodies (ezrin), autophagosomes (p62). Scale bars: 10 μm.
(TIFF)

S7 Fig Immunolabeling of Caco-2^*MYO5B−/−*^ cells. Vacuoles (asterisks) in Caco-2^*MYO5B*−/−^ were surrounded by microtubules (β-tubulin) and stained negative for markers of early/recycling endosomes (EEA1, TfR, rab8a, and rab11) but positive for markers of late endosome/lysosomes (rab7 and LAMP1). Endosomal markers were often found very close to the vacuoles (arrows), which could be because of their association with the perivacuolar microtubule cytoskeleton. To distinguish whether these markers were truly associated with the vacuoles or located subjacent to the vacuoles, we treated the cells with nocodazole (33 μM) for 2 h. In contrast to the resultant dispersal of early endosomal and recycling endosomal markers, rab7 and LAMP1 remained positive at the vacuoles. Scale bars: 10 μm. EEA1, early endosomal antigen-1; LAMP1, late endosome–associated membrane protein; TfR, transferrin receptor.
(TIFF)

S8 Fig Microscopical analyses of MVID tissue. (A) Electron microscopy of MVID patients’ duodenum. Black thick and thin arrows indicate microvillus inclusion bodies and electron-lucent vacuoles, respectively. (B–C) Immunohistochemistry of LAMP1 staining in MVID patients’ (B) and *MYO5B* KO mouse (C) duodenum and appropriate controls. Arrows indicate large LAMP1-positive vacuoles. Nuclei is stained with hematoxylin. KO, knockout; LAMP1, late endosome–associated membrane protein; MVID, microvillus inclusion disease.
(TIFF)

S9 Fig Loss of *MYO5B* induces large vacuoles and causes mitotic spindle orientation defects in HEK293 cells. (A) Western blotting analysis showed a complete loss of the myosin Vb protein in HEK293^*MYO5B−/−*^ cells. (B) Arrows indicate large vacuoles in HEK293^*MYO5B−/−*^ cells. The percentage of cells with vacuole (diameter Ø > 1 μm) was quantified in fixed HEK293^*WT*^ and HEK293^*MYO5B−/*−^ cells. (C) The images showed HEK293^*WT*^ cells, HEK293^*MYO5B−/*−^ cells without and with vacuoles stained as indicated in metaphase and anatelophase. The dotted circles in the images indicate large vacuoles. (D and E) The β-angle in metaphase (D) and anatelophase (E) was quantified in HEK293^*WT*^ and HEK293^*MYO5B−/*−^ cells. The dot graph: each dot indicates one cell’s β-angle. The histogram graph: the statistical analysis of the mean for each experiment. More than 10 cells/experiment were analyzed. (F and G) The β-angle in metaphase (F) and anatelophase (G) was quantified in HEK293^*MYO5B−/*−^ cells without and with vacuoles. *N* = 3 independent experiments. *t* test, *p < 0.05 **p < 0.01, ***p < 0.001, ****p < 0.0001. Error bars indicate ± SEM (dot graph) or + SD (bar graph). Scale bars: 5 μm. Values for each data point in panels B, D, E, F, and G can be found in S15 Data. HEK, human embryonic kidney cell; WT, wild type.
(TIFF)

S10 Fig Loss of *MYO5B* increases delamination and cytokinesis index in HEK293 cells. (A) The presence of cells not contacting the substratum was indicated by asterisks in nuclei. (B). The percentage of nonsubstrate-contacting cells was quantified. (C) Images showed the cytokinesis cells in HEK293^*MYO5B−/*−^ cells. (D) The percentage of cytokinesis cells was quantified in HEK293^*WT*^ and HEK293^*MYO5B−/*−^ cells. (E) The percentage of cytokinesis cells was quantified in HEK293^*MYO5B−/*−^ cells without and with vacuoles. *n* > 1,000 cells/experiment were analyzed for *N* = 3 independent experiments. *t* test, *p < 0.05, **p < 0.01. Error bars indicate + SD. Scale bars: 5 μm. Values for each data point in panels B, D, and E can be found in S16 Data. HEK, human embryonic kidney cell; ns, not significant; WT, wild type.
(TIFF)

S11 Fig Effect of rab7 and rab7 mutant on cytokinesis. (A) The percentage of cytokinesis was quantified in the fixed Caco-2^*MYO5B−/−*^ cells expressing GFP-rab7-WT or GFP-rab7-T22N. (B) The percentage of cytokinesis was quantified in the fixed Caco-2^*MYO5B−/−*^ cells expressing GFP-rab7-T22N with and without vacuoles. *N* = 3 independent experiments. *t* test, error bars indicate +SD. Values for each data point can be found in S17 Data. GFP, green fluorescent protein; ns, not significant; WT, wild type.
(TIFF)

S1 Table List of antibodies used. (DOCX)

S1 Video Live cell imaging shows cytokinesis duration in Caco-2^*WT*^ cells expressing β-tubulin-GFP and histone2B-mCherry. Arrow in the video indicates the cytokinesis bridge. The time of cytokinesis duration was compared as shown in Fig 2B and 2C. Scale bar and duration time are shown in the lower left corner and lower right corner in the video, respectively. GFP, green fluorescent protein; WT, wild type.
(MP4)

S2 Video Live cell imaging shows cytokinesis duration in Caco-2^*MYO5B−/−*^ cells expressing β-tubulin-GFP and histone2B-mCherry. Arrow in the video indicates the cytokinesis bridge. The time of cytokinesis duration was compared as shown in Fig 2B and 2C. Scale bar and duration time are shown in the lower left corner and lower right corner in the video, respectively. GFP, green fluorescent protein.
(MP4)

S3 Video Live cell imaging shows spindle rotation in Caco-2^*MYO5B−/−*^ cells expressing β-tubulin-GFP and histone2B-mCherry without a vacuole. The spindle rotation angle was compared as shown in Fig 4A and 4B. Scale bar and duration time are shown in the lower left corner and lower right corner in the video, respectively. GFP, green fluorescent protein.
(MP4)

S4 Video Live cell imaging shows spindle rotation in Caco-2^*MYO5B−/−*^ cells expressing β-tubulin-GFP and histone2B-mCherry with a vacuole. White circle indicates the vacuole. The spindle rotation angle was compared as shown in Fig 4A and 4B. Scale bar and duration time are shown in the lower left corner and lower right corner in the video, respectively. GFP, green fluorescent protein.
(MP4)

S5 Video Live cell imaging shows spindle rotation in Caco-2^*MYO5B−/−*^ cells expressing β-tubulin-GFP and histone2B-mCherry with a vacuole. White circle in the video indicates the vacuole. Red dot indicates the spindle pole. The distance from spindle pole to vacuole in the onset and end of metaphase was compared as shown in Fig 4C–4E. Scale bar and duration time are shown in the lower left corner and lower right corner in the video, respectively. GFP, green fluorescent protein.
(MP4)

S6 Video Live cell im .aging shows metaphase arrest in Caco-2^*MYO5B−/−*^ cells expressing β-tubulin-GFP and histone2B-mCherry with a vacuole (shown in S4B Fig). White circle in the video indicates the vacuole. The duration of metaphase time in this cell was more than 5 h. Scale bar and duration time are shown in the lower left corner and lower right corner in the video, respectively. GFP, green fluorescent protein.
(MP4)

S1 Data Values for each data point used to create the graphs in Fig 1. (XLSX)

S2 Data Values for each data point used to create the graphs in Fig 2. (XLSX)

S3 Data Values for each data point used to create the graphs in Fig 3. (XLSX)

S4 Data Values for each data point used to create the graphs in Fig 4. (XLSX)

S5 Data Values for each data point used to create the graphs in Fig 5. (XLSX)

S6 Data Values for each data point used to create the graphs in Fig 6. (XLSX)

S7 Data Values for each data point used to create the graphs in Fig 7. (XLSX)

S8 Data Values for each data point used to create the graphs in Fig 8. (XLSX)

S9 Data Values for each data point used to create the graphs in Fig 9. (XLSX)

S10 Data Values for each data point used to create the graphs in Fig 10. (XLSX)

S11 Data Values for each data point used to create the graphs in S2 Fig. (XLSX)

S12 Data Values for each data point used to create the graphs in S3 Fig. (XLSX)

S13 Data Values for each data point used to create the graphs in S4 Fig. (XLSX)

S14 Data Values for each data point used to create the graphs in S5 Fig. (XLSX)

S15 Data Values for each data point used to create the graphs in S9 Fig. (XLSX)

S16 Data Values for each data point used to create the graphs in S10 Fig. (XLSX)

S17 Data Values for each data point used to create the graphs in S11 Fig. (XLSX)

## Abbreviations

ANO: anoctamin
BafA1: bafilomycin A1
DIDS: 4,4'-Diisothiocyano-2,2'-stilbenedisulfonic acid
DN: dominant negative
eGFP: enhanced green fluorescent protein
GFP: green fluorescent protein
HEK: human embryonic kidney cell
KO: knockout
LAMP: late endosome–associated membrane protein
LC: light chain
MVID: microvillus inclusion disease
NPPB: 5-nitro-2-(3-phenylpropyl-amino) benzoic acid
vATPase: vacuolar H^+^-adenosine triphosphatase
Wm: wortmannin
WT: wild type
