# Supplementary material for: Loss of MYO5B expression deregulates late endosome size which hinders mitotic spindle orientation
Source: PLoS Biol. 2019 Nov 4;17(11):e3000531. doi: 10.1371/journal.pbio.3000531 (PMC6855566; doi:10.1371/journal.pbio.3000531)
Supplement: S1 Table — (DOCX) [file pbio.3000531.s012.docx]

**S1 Supplementary table 1**

**List of antibodies used in this study.**

| **Antibody target** | **Species** | **Manufacturer** | **Catalog number** | **Dilution** |
| --- | --- | --- | --- | --- |
| Rab7 | Rabbit | Cell signaling | 2094 | 1:200 |
| LAMP1 | Mouse | DSHB | H4A3 | 1:200 |
| EEA1 | Mouse | Abcam | ab70521 | 1:200 |
| Transferrin receptor | Mouse | Invitrogen | 13-6800 | 1:200 |
| Rab8a | Mouse | Abnova | M02 | 1:200 |
| Rab11 | Mouse | Biosciences | 610656 | 1:200 |
| Giantin | Rabbit | Biolegend | PRB114C | 1:200 |
| Calnexin | Rabbit | StressGen | SPA-860 | 1:200 |
| Ezrin | Rabbit | Santa Cruz | sc-20773 | 1:200 |
| LC-3B | Mouse | Nanotools | 0231-100 | 1:50 |
| P62 | Guinea Pig | Progen | GP62-C | 1:200 |
| β-Tubulin | Mouse | Sigma | T4026 | 1:200 |
| ZO-1 | Mouse | Thermo | 33-9100 | 1:200 |
| ZO-1 | Rabbit | Thermo | 40-2200 | 1:200 |
| LAMP1 | Rat | Santa Cruz | sc-19992 | 1:50 |
| Na^+^/K^+^ ATPase | Mouse | Santa Cruz | sc-21712 | 1:200 |
| C-myc | Mouse | Clontech | 631206 | 1:200  1:1000 WB |
| Myosin Vb | Rabbit | NOVUS | NBP1-87746 | 1:1000 WB |
| β-Actin | Mouse | Sigma | A5441 | 1:1000 WB |
